# Chick cranial neural crest cells release extracellular vesicles that are critical for their migration

**DOI:** 10.1242/jcs.260272

**Published:** 2022-06-28

**Authors:** Callie M. Gustafson, Julaine Roffers-Agarwal, Laura S. Gammill

**Affiliations:** 1 Department of Genetics, Cell Biology and Development, University of Minnesota, 6-160 Jackson Hall, 321 Church St SE, Minneapolis, MN 55455, USA; 2 Developmental Biology Center, University of Minnesota, 6-160 Jackson Hall, 321 Church St SE, Minneapolis, MN 55455, USA

**Keywords:** Neural crest, Migration, Extracellular vesicles, Exosomes, Migrasomes

## Abstract

The content and activity of extracellular vesicles purified from cell culture media or bodily fluids have been studied extensively; however, the physiological relevance of exosomes within normal biological systems is poorly characterized, particularly during development**.** Although exosomes released by invasive metastatic cells alter migration of neighboring cells in culture, it is unclear whether cancer cells misappropriate exosomes released by healthy differentiated cells or reactivate dormant developmental programs that include exosome cell–cell communication. Using chick cranial neural fold cultures, we show that migratory neural crest cells, a developmentally critical cell type and model for metastasis, release and deposit CD63-positive 30–100 nm particles into the extracellular environment. Neural crest cells contain ceramide-rich multivesicular bodies and produce larger vesicles positive for migrasome markers as well. We conclude that neural crest cells produce extracellular vesicles including exosomes and migrasomes. When Rab27a plasma membrane docking is inhibited, neural crest cells become less polarized and rounded, leading to a loss of directional migration and reduced speed. These results indicate that neural crest cell exosome release is critical for migration.

## Abstract (precis)

**Summary:** Extracellular vesicles are well defined in cancer, but their normal functions remain less certain. As they migrate, chick neural crest cells deposit migrasomes and release exosomes that are essential for motility.

## INTRODUCTION

Exosomes are small (30–100 nm), membrane-bound particles that are released from cells to mediate autocrine, paracrine and endocrine cell–cell communication (Sung et al., 2021). Although small, exosomes carry powerful messages that prompt large scale changes in the cells around them, making them a popular topic of interest in research (Ale Ebrahim et al., 2020). Their cargo includes bioactive molecules and materials like lipids (ceramides and sphingomyelin), membrane and cytosolic proteins (tetraspanins, integrins and signaling receptors) and nucleic acids (mRNA and microRNAs) (Gurung et al., 2021). Although many cell types release exosomes, and all cells are assumed to, the function and physiological relevance of exosomes within normal biological systems are poorly characterized (Kalluri and LeBleu, 2020).

Exosomes are heavily studied in cancer, where they have a number of crucial functions (McAndrews and Kalluri, 2019). Invasive metastatic cancer cells produce exosomes to induce an epithelial-to-mesenchymal transition (EMT) in surrounding cells (Conigliaro and Cicchini, 2018; Xiao et al., 2016), influence distant cell types to condition the pre-metastatic niche (Peinado et al., 2016) and change migration behaviors of neighboring cells in culture (Qiao et al., 2019; Steenbeek et al., 2018; Sung et al., 2021, 2020; Zomer et al., 2015). Moreover, highly metastatic melanoma cells release exosomes with distinct proteomic signatures (Lazar et al., 2015) that transfer their metastatic capacity and motility to less invasive melanoma cells (Felicetti et al., 2016). Cancer exosome research, largely performed on cell lines *in vitro*, yields enormous promise for innovative drug delivery, identification of diagnostic biomarkers and discovery of cancer mechanisms (Pullan et al., 2019; Soung et al., 2017; Tickner et al., 2014). The issue remains, however, that although cultured cancer cells are useful for isolating and studying large quantities of exosomes, comparatively less is known about how exosomes function in a normal physiological context where exosome release is not dysregulated (Kalluri and LeBleu, 2020). It is unclear whether cancer cells ‘weaponize’ exosomes produced normally by healthy functioning cells or reactivate dormant developmental programs that require exosome release. This knowledge is particularly lacking because research on embryos and complex multicellular structures is difficult to adapt to exosome cell culture protocols (McGough and Vincent, 2016).

Neural crest cells (NCCs) offer a unique system to study cancer-relevant mechanisms in a normal developmental context (Gallik et al., 2017). NCCs are transient, multipotent and migratory cells that travel widely across the early embryonic landscape to form diverse vertebrate-specific structures (Dash and Trainor, 2020; Martik and Bronner, 2021). Like metastatic cells leaving a solid cancerous tumor, NCCs undergo EMT and migrate away from their site of origin in the dorsal neural tube using related transcriptional, signaling and morphological processes (Powell et al., 2013; Theveneau and Mayor, 2012). As a cancer of NCC-derived melanocytes, melanoma cells reactivate the NCC transcriptional program (Capparelli et al., 2022; Kaufman et al., 2016; Rambow et al., 2018) and follow environmental cues to migrate along NCC pathways (Bailey et al., 2012), suggesting that NCCs migrate and melanoma cells metastasize using shared mechanisms.

Cranial NCCs migrate as dense multicellular streams with defined leader and trailer cells (McLennan et al., 2015a; Wynn et al., 2013). NCC streams are organized by chemotaxis and an assortment of physical and molecular environmental guidance cues (Shellard et al., 2018). Within the stream, the trajectory of movement polarizes protrusive activity, which leads to directed migration of individualized chick cranial NCCs (Genuth et al., 2018; Kulesa et al., 2010, 2008), whereas collectively migrating *Xenopus* NCCs also achieve directed movement through contact-inhibition-of-locomotion (Carmona-Fontaine et al., 2008; Theveneau et al., 2010). As they migrate and explore their environment, NCCs communicate with each other through cell contact (Piacentino et al., 2020) and by direct exchange of information via thin filopodial extensions (McKinney et al., 2011; Teddy and Kulesa, 2004) and gap junctions (Huang et al., 1998; Jourdeuil and Taneyhill, 2020). Moreover, NCCs undergo macropinocytosis and take up material from the environment (Li et al., 2020; Padmanabhan and Taneyhill, 2015). Tumor cells shed exosomes that induce chemotaxis and directional migration of adjacent cells (Steenbeek et al., 2018; Sung et al., 2015; Sung and Weaver, 2017; Zomer et al., 2015); however, no evidence currently exists on whether the material or information exchanged by migratory NCC might include exosomes, and whether extracellular vesicles regulate NCC migration, despite the similarities of NCC behaviors to cancer.

In this paper, we show that chick migratory cranial NCC release extracellular vesicles with the size and characteristics of exosomes and migrasomes. Inhibiting exosome release using the Rab27a inhibitor Nexinhib20 (Johnson et al., 2016; Zhang et al., 2020) disrupts NCC migration and morphology, yielding smaller, rounder and slower NCCs. We conclude from our data that extracellular vesicles are required for NCC migration.

## RESULTS

### Cranial NCCs release and deposit CD63-positive vesicles into the extracellular environment

We became interested in the possibility that NCCs secrete exosomes after finding that tetraspanin18 (Tspan18) antagonizes NCC EMT (Fairchild et al., 2014; Fairchild and Gammill, 2013), as Tspans are highly enriched in exosomes (van Niel et al., 2018). To determine which Tspans in addition to Tspan18 are expressed by NCCs, we evaluated RNA-seq data from chick premigratory cranial NCCs (Jacques-Fricke et al., 2021). Among the 30 member Tspan family, CD63 was the most abundant transcript, 1.8-fold more prevalent than the next highest Tspan. CD63 is a well-characterized canonical exosome marker that accumulates in multivesicular bodies (MVBs) and exosomes (Jeppesen et al., 2019) and has known roles in intracellular trafficking, cargo sorting and exosome formation (Justo and Jasiulionis, 2021; van Niel et al., 2015, 2011). Thus, CD63 expression led us to postulate that NCCs produce exosomes to regulate their development.

We first set out to visualize NCC exosome release. We used an optical reporter that includes the full-length human CD63 protein fused to the pH-sensitive GFP moiety pHluorin (Sung et al., 2015; Verweij et al., 2018). When this fusion protein is expressed, CD63 localizes pHluorin to the exosome pathway. The acidity of endosomal compartments, where exosome biogenesis occurs, quenches pHluorin fluorescence. When MVBs fuse with the plasma membrane, the neutral pH stabilizes pHluorin, fluorescently indicating exosome release (Bebelman et al., 2020; Sung et al., 2021; Verweij et al., 2018).

**Fig. 1.**
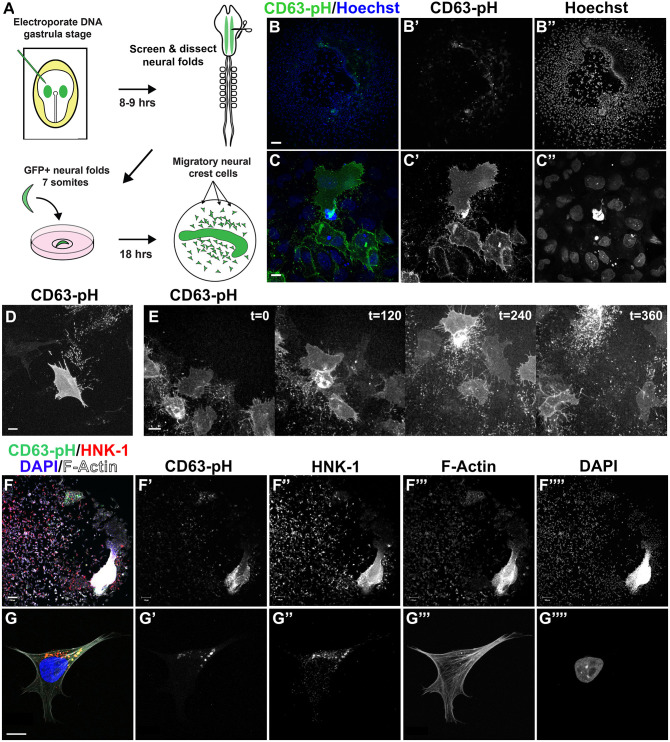
**Cranial NCCs release and deposit CD63-positive vesicles into the extracellular environment.** (A) Experimental workflow for electroporation, dissection, and culture of NCCs from neural fold explants. (B,C) Live neural fold cultures (B–B″) and migratory NCCs (C–C″) expressing pCMV-CD63-pHluorin (CD63–pH; exosomes, green) and stained with Hoechst 33342 (nucleus, blue). Scale bars: 100 µm (B); 10 µm (C). (D) Live NCC releasing a trail of CD63–pH-positive exosome deposits behind it. Scale bar: 10 µm. (E) 3 h time lapse frames (*t*=min) of cultured CD63–pH-expressing NCCs. Images represent maximum intensity projections. Scale bar: 10 µm. (F,G) Fixed neural fold cultures (F–F″″) and an individual NCC (G–G″″) expressing CD63–pH (green) and stained for HNK-1 (migratory NCCs, red), F-actin (cytoskeleton, white) and DAPI (nucleus, blue). Scale bars: 100 µm (F); 10 µm (G). Images are representative of three experiments.

We combined this reporter of exosome secretion with chick primary NCC cultures. Although NCCs normally migrate deep within the complex three-dimensional embryonic environment, imaging exosome release in this context presents technical and imaging resolution challenges. However, NCCs emigrate from cranial neural fold explants in culture, allowing detailed analysis of individual cells recently and naturally segregated from an embryonic tissue context (Bronner-Fraser and Garcia-Castro, 2008). Gastrula stage chick embryos were bilaterally electroporated (Gammill et al., 2019) with pCMV-CD63-pHluorin (CD63–pH) (Verweij et al., 2018) and re-incubated until the 6–7 somite stage. CD63–pH-positive cranial neural folds were carefully dissected, plated on fibronectin and incubated for 18 h (Fig. 1A). Expression of CD63–pH was mosaic across the culture (Fig. 1B–B″), and individual electroporated migratory NCC showed plasma membrane fluorescence along with bright moving puncta in the extracellular space (Fig. 1C–C″; Movie 1), similar to what has been observed in previous studies of other cell types (Sung et al., 2015; Verweij et al., 2018). In addition to releasing exosomes that moved freely in the extracellular environment, CD63–pH-positive NCCs also left behind exosome trails or deposits (Fig. 1D,E; Movie 2). Although CD63–pH fluoresces only after plasma membrane fusion in live cultures, fixation neutralizes cellular pH to reveal pHluorin within cells (Sung et al., 2020). After fixation, although extracellular structures were not retained, CD63–pH was observed within NCCs in round endosomal structures that co-stained for the migratory NCC marker HNK-1 (Chou et al., 1986; Tucker et al., 1984) (Fig. 1F–F″″,G–G″″), further supporting exosome biogenesis taking place in HNK-1-positive NCCs. These results show that NCCs release and deposit CD63–pH-positive vesicles that behave in a manner expected of exosomes.

### Cranial NCCs contain ceramide-rich MVBs and generate migrasomes

Because the CD63–pH reporter could elicit exosome secretion as an overexpression artifact, we also wanted to visualize NCC exosome biogenesis without exogenous expression of CD63. Since Tspans are small four-pass transmembrane proteins, and antibodies raised against them recognize the divergent extracellular loops (Lang and Hochheimer, 2020), commercial Tspan antibodies, including those raised against CD63, do not cross-react with chick. Instead, we evaluated other proteins that are used to validate the presence of exosomes in purified preparations, like the heat-shock cognate protein Hsc70 (Jeppesen et al., 2019; Théry et al., 2018). Unfortunately, Hsc70 is a cytoplasmic housekeeping protein that is ubiquitously expressed, with numerous chaperone functions (Daugaard et al., 2007). As a result, Hsc70 is distributed throughout the cytoplasm, as seen in NCCs (Fig. S1), and is not a marker of exosome transport or biogenesis in whole cells. Also, we could not visualize Hsc70 in soluble exosomes since they are removed with the media for fixation and staining.

**Fig. 2.**
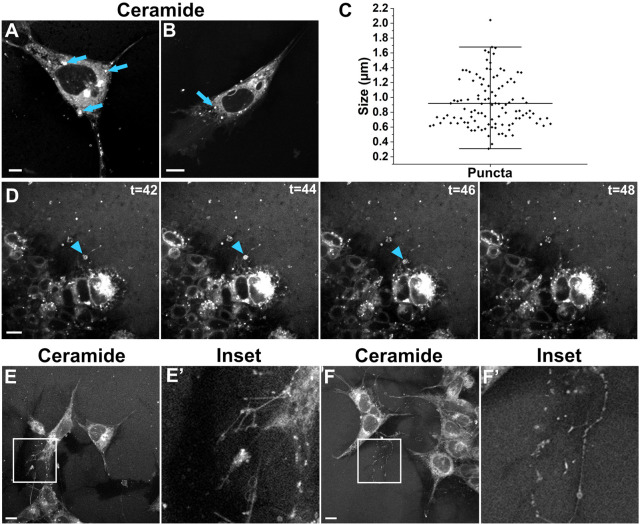
**Cranial NCCs contain ceramide-rich MVBs and produce or take up large extracellular vesicles.** (A,B) Cultured live NCCs stained with BODIPY ceramide to reveal MVBs (brightly fluorescent puncta, blue arrows). Scale bars: 10 µm (A,B). (C) Scatter plot distribution of measured puncta diameter, average 0.92±0.33 µm, mean±s.d.; *n*=111. (D) Time lapse frames (*t*=min) of BODIPY ceramide-stained NCCs as they take up a nearby migrasome (blue arrowheads). Scale bar: 20 µm. (E,F) BODIPY ceramide-stained NCCS with inset panels (E′,F′) to highlight intact and degrading retraction fibers. Scale bar: 10 µm. Images in D–F′ are representative of five experiments.

Since exosome biogenesis is a ceramide-dependent process (Trajkovic et al., 2008), cells incorporate fluorescently labeled ceramides into exosomes as they form in MVBs, allowing for visualization of MVBs and exosomes in culture (Laulagnier et al., 2005). Within and surrounding NCCs treated with BODIPY TR ceramide (BODIPY ceramide) brightly fluorescent, ceramide-rich moving puncta were apparent, indicating the presence of MVBs and exosome biogenesis (Fig. 2A,B; Movies 3,4). This indicates that native migratory NCCs produce exosomes.

As we imaged BODIPY ceramide-stained NCCs at higher magnification, we noticed a variety of labeled extracellular structures. Although similar to CD63–pH exosome deposits (Fig. 1D), some of these larger puncta were 300 nm–2.0 µm in diameter (Fig. 2C), suggesting production of migrasomes. Bigger in size than other extracellular vesicles, migrasomes form on tetraspanin-enriched retraction fibers left behind migrating cells (Huang et al., 2019). As the retraction fibers degrade, the migrasomes can be taken up by surrounding cells (Ma et al., 2015), which we also observed in a time lapse of NCCs stained with BODIPY ceramide (Fig. 2D; Movie 4). In addition to tiny puncta indicative of exosomes (Laulagnier et al., 2005), cells left behind very thin filamentous, branching fibers attached to larger, round puncta (Fig. 2E), as well as degraded retraction fibers (Fig. 2F).

**Fig. 3.**
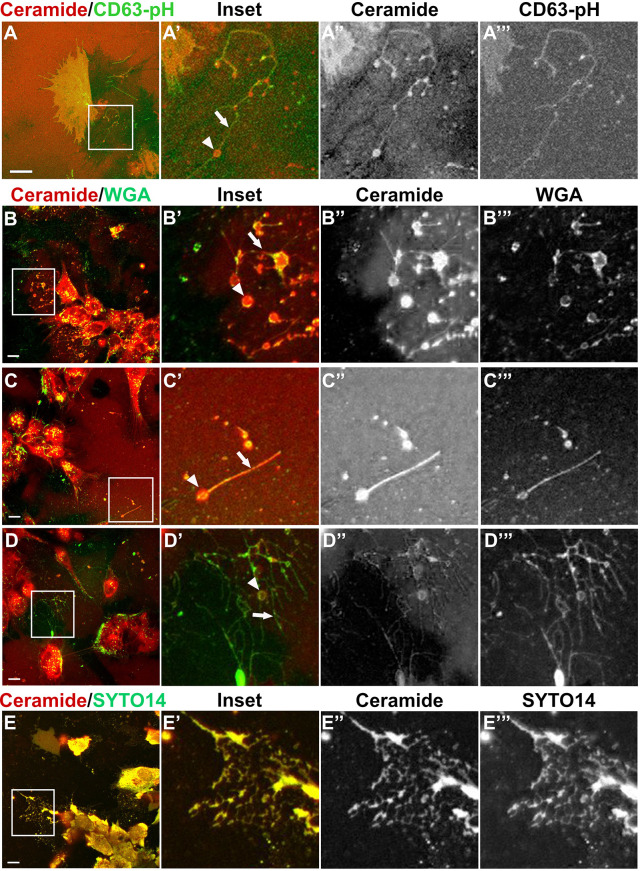
**Large extracellular vesicles released from NCCs are positive for multiple migrasome markers.** (A) An NCC (A) expressing CD63–pH and stained with BODIPY ceramide with an inset panel (A′–A‴) to highlight a migrasome (white arrowheads) and retraction fiber (white arrow). Scale bar: 10 µm. (B–D) BODIPY ceramide and WGA co-stained NCCs with inset panels (B′–B‴,C′–C‴,D′–D‴) to show the colocalization of BODIPY ceramide and WGA in migrasomes (white arrowheads) and retraction fibers (white arrows). Scale bars: 10 µm. (E) NCCs stained with BODIPY ceramide and SYTO14, with inset panels to show colocalization (E′–E‴). Scale bars: 10 µm. Images are representative of three experiments.

To confirm the presence of migrasomes in live NCC cultures, we used previously identified migrasome stains (Chen et al., 2019; Zhu et al., 2021). Migrasomes contain CD63 (Zhu et al., 2021), which was observed in NCCs expressing low levels of CD63–pH and stained with BODIPY ceramide (Fig. 3A). In this case, retraction fibers were particularly enriched with CD63–pH whereas migrasomes were more brightly labeled with BODIPY ceramide (Fig. 3A′–A‴). Although CD63 does not promote migrasome formation (Huang et al., 2019), to confirm that NCCs release migrasomes without Tspan overexpression, we also stained cells with wheat-germ agglutinin (WGA), which preferentially binds to migrasomes over retraction fibers (Chen et al., 2019). NCC cultures contained large extracellular vesicles brightly positive for BODIPY ceramide and WGA in branching networks of retraction fibers (Fig. 3B–B‴,D–D‴) and singular ball and stick-like structures (Fig. 3C–C‴). As a secondary marker of migrasomes, we also stained NCC with the nucleic acid stain SYTO14, which labels mRNA present within migrasomes (Zhu et al., 2021). While the signal to noise was not ideal compared to the WGA staining, colocalization of BODIPY ceramide and SYTO14 in large extracellular vesicles was observed in NCC cultures (Fig. 3E–E‴). These results show that in addition to generating ceramide-rich MVB and extracellular puncta consistent with exosomes, NCC leave behind trails of nucleic acid-containing migrasomes. These data also indicate that, similar to WGA, BODIPY ceramide can be used as an alternate or additional migrasome stain.

### NCCs release exosome-sized particles

**Fig. 4.**
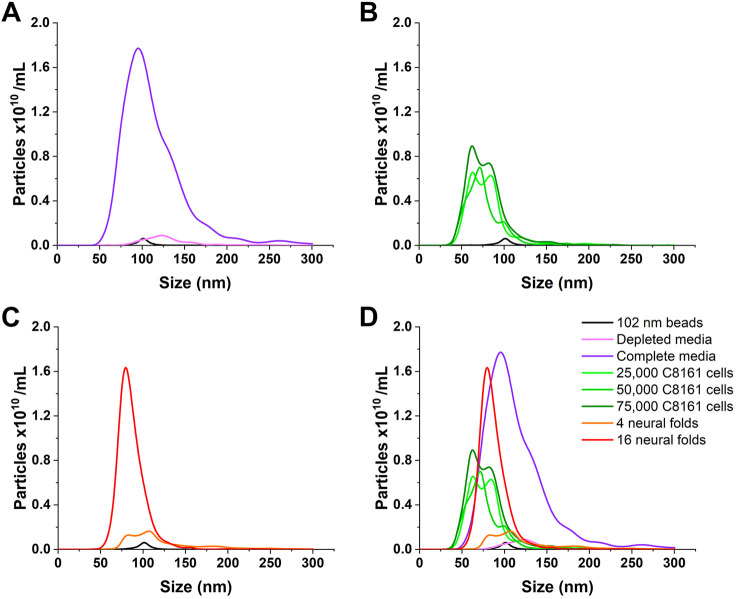
**NCCs release exosome-sized particles.** Nanoparticle Tracking Analysis of particle size (nm) and concentration (particles×10^10^/ml) in neural fold and C8161 cell culture media in comparison to a 102 nm polystyrene bead standard. (A) Complete neural crest culture medium versus medium depleted of exosomes (30–100 nm) and other small particles by ultracentrifugation at 100,000 ***g***. (B) Depleted medium cultured with 25,000, 50,000 and 75,000 C8161 human melanoma cells. (C) Depleted medium cultured with 4 and 16 neural folds. (D) Merged graphs of all experimental conditions. Each trace represents data averaged from five replicate video captures of one biological experiment.

Although the CD63 reporter and lipid marker indicate the presence of exosomes, we also wanted to ensure that NCC-released particles were the size of exosomes. Nanoparticle tracking analysis (NTA) uses light scattering imaging and proprietary software to detect nanosized (10–1000 nm) particles in solution. NTA provides size and concentration data to distinguish between various types of cell-derived extracellular vesicles (Dragovic et al., 2011). We first used ultracentrifugation to deplete exosomes normally present in NCC complete culture medium due to the use of chick embryo extract and fetal bovine serum (Shelke et al., 2014), dramatically reducing particles in the 30–100 nm range (Fig. 4A). NCCs migrated normally from neural folds in exosome-depleted culture medium, so we used this media in all of our cultures for NTA analysis (Fig. S2A). As a positive control, we collected media cultured with NCC-derived C8161 human melanoma cells. As previously described, C8161 cells produce large quantities of exosomes (Felicetti et al., 2009), apparent by NTA as particles in the 30–100 nm size range (Fig. 4B,D). Meanwhile, media cultured with 4 or 16 neural folds contained 30–100 nm-sized particles at a concentration comparable to or greater than C8161 cells (Fig. 4C,D; Fig. S2B,C), demonstrating that NCCs release exosome-sized particles, similar to invasive melanoma cells.

### Inhibiting exosome secretion disrupts NCC migration and morphology

**Fig. 5.**
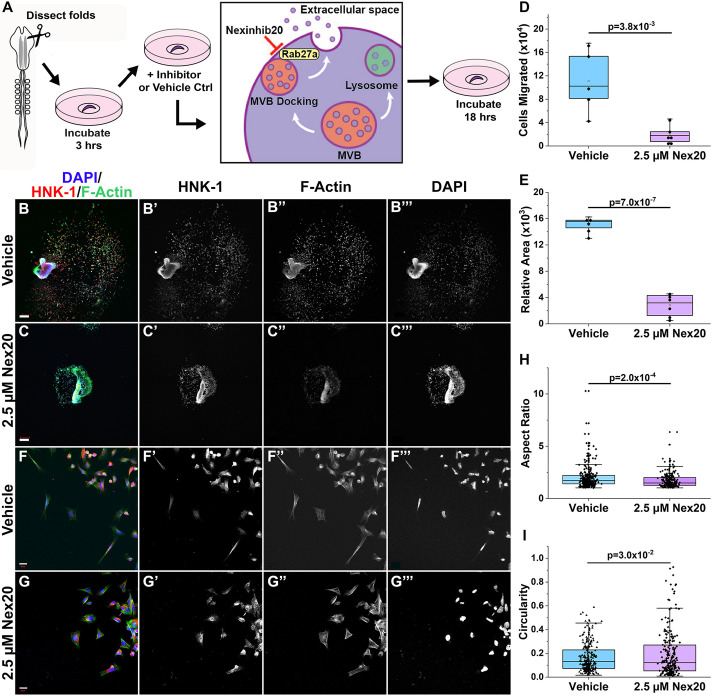
**Inhibiting exosome secretion disrupts NCC migration and morphology.** (A) Workflow for Nexinhib20 (Nex20) treatment of NCCs prior to imaging and analysis. Diagram illustrates Nex20 inhibition of Rab27a, which prevents MVBs from docking to the plasma membrane and releasing exosomes into the extracellular space. (B–I) Vehicle (B–B‴,F–F‴) and 2.5 µM Nex20-treated (C–C‴,G–G‴) neural fold cultures (B,C) or NCCs (F,G) stained for HNK-1 (migratory NCCs, red), F-actin (cytoskeleton, green) and DAPI (nucleus, blue). (D,E) Box-and-whisker plots showing area occupied by migrating cells (D; *P*=3.8×10^−3^) and total number of cells migrated (E; *P*=7.0×10^−7^) in vehicle (*n*=5 folds) and 2.5 µM Nex20 (*n*=6 folds)-treated neural fold cultures. (H,I) Box and whisker plots showing aspect ratio (H; *P*=2.0×10^−4^) and circularity (I; *P*=3.0×10^−2^) in vehicle (*n*=252 cells) and 2.5 µM Nex20 (*n*=223 cells)-treated NCCs. Scale bars: 100 µm (B,C); 10 µm (F,G). For the box-and-whisker plots, the box represents the 25–75th percentiles, and the mean is indicated. The whiskers show the 10th and 90th percentile outlier range. All *P*-values were calculated with an unpaired one--tailed Student's *t*-test.

To test whether exosome secretion is required for NCC migration, we blocked the final step in exosome biogenesis – docking of Rab27a on MVBs to a synaptotagmin-like homology domain (SHD) protein at the plasma membrane (Fukuda, 2013). This step is necessary for MVB fusion but does not affect other secretory pathways (Ostrowski et al., 2010). Although we normally disrupt gene function in chick NCC using antisense morpholino oligonucleotides, introduction of reagents into neural folds by electroporation is mosaic (see for example Fig. 1B). Since exosomes are shed into the environment and can act non-cell autonomously, cells with sub-optimal morpholino delivery in the culture would be expected to neutralize any knockdown effect. Thus, to acutely and uniformly inhibit Rab27a docking in the entire migratory cranial NCC population, we utilized the small molecule inhibitor Nexinhib20 (Nex20), which specifically disrupts Rab27a–SHD interaction without interfering with other Rabs to prevent exosome release (Johnson et al., 2016; Zhang et al., 2020) (Fig. 5A). To remove any effect of exosomes provided in complete media, we diluted Nex20 in exosome-depleted medium (Fig. 4A). Neural folds from 7 somite embryos were dissected and plated on fibronectin in 2.5 µM Nex20, then incubated overnight. Interestingly, neural folds failed to adhere to fibronectin in the presence of Nex20, consistent with the ability of Nex20 to inhibit exocytic surface presentation of adhesion molecules (including an integrin) following neutrophil activation (Johnson et al., 2016). As a result, to analyze active migration, 7 somite embryo neural folds were dissected and allowed to adhere to fibronectin for 3 h (9 somite embryo equivalent) before replacing the complete medium with 2.5 µM Nex20 in exosome-depleted medium and incubating overnight (Fig. 5A). Cultures were fixed and stained for HNK-1, DAPI and F-actin to visualize NCC, the nucleus and cytoskeleton, respectively. Nex20-treated NCC cultures (*n*=6) were smaller with less dispersed NCCs compared to vehicle controls (*n*=5) (Fig. 5B–B‴,C–C‴). There was a significant (*P*=3.8×10^−3^) decrease in the number of cells that migrated (Fig. 5D) as well as the relative area (*P*=7×10^−7^) that NCCs occupied on the coverslip (Fig. 5E). As cell death would also reduce NCC number, TUNEL TMR staining was performed and cells were counted in untreated (*n*=2041), vehicle (*n*=5010) and 2.5 µM Nex20-treated (*n*=4231) cultures. The proportion of dying cells did not differ significantly between treatment groups, averaging 4% TUNEL-positive cells (Fig. S3A–D; *P*=1.00). These data show that fewer NCCs disperse from cranial neural folds after Nex20 treatment, indicating that exosome release is necessary for NCC migration.

Upon examination at higher magnification, individual Nex20-treated NCC exhibited altered morphology (Fig. 5F–F‴,G–G″). Whereas the actin cytoskeleton of vehicle-treated NCCs (*n*=252) was polarized and elongated (Fig. 5F″), Nex20-treated NCCs (*n*=223) were more compact and rounded (Fig. 5G″). To quantify this change in cell shape, we measured the length (L) and width (W) of individual NCC, referred to as the aspect ratio (L/W), where a value closer to 1 indicates a less polarized cell as the L and W become the same. Nex20-treated NCCs had a smaller aspect ratio (1.7±0.7; mean±s.d.) compared to vehicle (2.0±1.0; Fig. 5H; *P*=2.0×10^−4^). We also measured circularity, which takes into account the presence of filopodia, with a value closer to 1 being more circular and less protrusive. Nex20-treated NCC also exhibited greater circularity (0.19±0.1 compared to 0.16±0.2 for vehicle; Fig. 5I; mean±s.d.; *P*=0.0303). Together, these results reveal that Nex20-treated NCCs are less polarized and less protrusive, either of which affect their motility (McLennan and Kulesa, 2010). We obtained similar (although less dramatic) disruption of NCC migration and cell shape with the commonly used exosome inhibitor GW4869; however, GW4869 blocks only one exosome biogenesis pathway [endosomal sorting complexes required for transport (ESCRT)-mediated exosome secretion] (Trajkovic et al., 2008). Moreover, GW4869 exosome specificity was recently questioned (Mathieu et al., 2021) and the DMSO vehicle caused NCC cell death (data not shown). In addition, we attempted to rescue migration by performing Nex20 treatment in media pre-conditioned with 16 neural folds (as in Fig. 4C). However, any improvement was impossible to discern from normal variation in neural fold cultures (data not shown).

### Rab27a inhibition affects NCC motility and exosome release

**Fig. 6.**
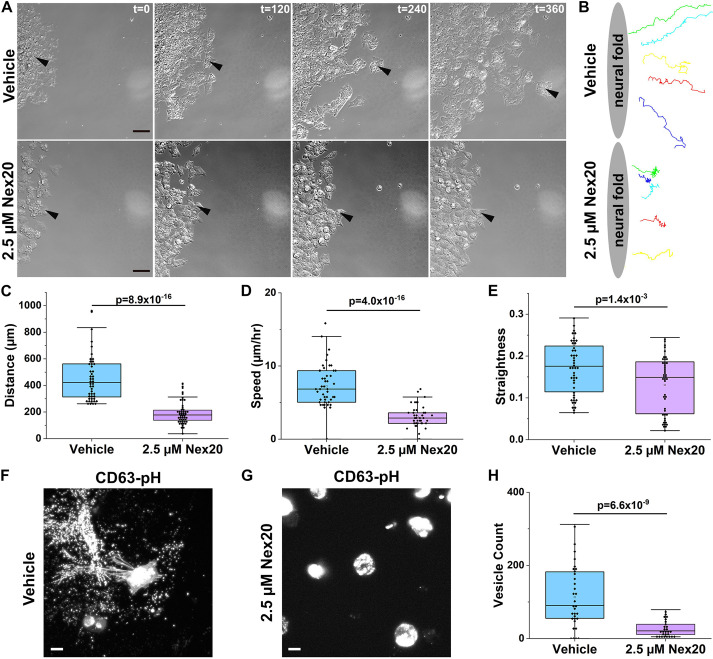
**Rab27a inhibition affects NCC motility and exosome release.** (A) Time lapse imaging frames (*t*=min) of neural crest cultures treated with vehicle or 2.5 µM Nex20. Black arrowheads point to single NCCs followed over the course of 6 h as each reaches its furthest point away from the neural fold. Images represent maximum intensity projections. Scale bars: 40 µm. (B) NCCs were manually tracked using MTrackJ plugin in ImageJ. Representative tracks of vehicle and 2.5 µM Nex20-treated NCCs from one of three biological replicate experiments were plotted. (C–E) Box-and-whisker plot comparison of vehicle or 2.5 µM Nex20-treated individual NCCs tracked in ImageJ (vehicle *n*=45; 2.5 µM Nex20 *n*=45). (C) Distance was measured as the total distance traveled between the first and last frame capture from t=0 to t=6 h (*P*=8.9×10^−16^). (D) Speed was determined as the total distance traveled over the 6 h time course (*P*=4.0×10^−16^). (E) Straightness was calculated as a ratio of the total displacement distance (µm) of the tracked NCC from the first and last frame of the recording over the total distance traveled (µm) where the maximum straightness would be a value of 1 (*P*=1.4×10^−3^). (F,G) Live CD63–pH expressing NCCs 1 h post-treatment with vehicle (F) or 2.5 µM Nex20 (G). Scale bars: 10 µm. (H) Box-and-whisker plot comparison of counted vesicles from vehicle or 2.5 µM Nex20-treated cells from F and G using the Analyze Particle function in ImageJ (vehicle *n*=25 fields of cells from 6 folds; 2.5 µM Nex20 *n*=28 fields of cells from 6 folds; Student's *t*-test, *P*=6.6×10^−9^). Data represents three biological replicates. For the box-and-whisker plots, the box represents the 25–75th percentiles, and the mean is indicated. The whiskers show the 10th and 90th percentile outlier range. All *P*-values were calculated with an unpaired one-tailed Student's *t*-test.

To characterize the effect of Rab27a inhibition on NCC motility, we imaged Nex20-treated NCCs over time. Neural folds from 7 somite embryos were dissected and incubated in complete medium for 4 h (9–10 somite embryo equivalent) so that NCCs had migrated far enough to be easily distinguished from one another. After addition of 2.5 µM Nex20 in exosome-depleted medium, NCCs at the migratory front were imaged every 2 min for 6 h (Fig. 6A; Movie 5). As quantified in fixed samples (Fig. 5), Nex20-treated cells did not migrate as far and appeared to be rounder and less polarized than vehicle controls (Fig. 6A). Tracks of individual Nex20-treated NCCs were shorter and exhibited wandering behavior compared to vehicle-treated NCCs, with movement away from the fold evident only early in the time course (likely reflecting the time it takes for Rab27a inhibition to take effect; Fig. 6B). Indeed, Nex20-treated NCCs traveled significantly less distance (*n*=45; 190.2±79.6 µm; mean±s.d.) compared to vehicle NCC (*n*=45; 449.8±161.8 µm; Fig. 6C; *P*=8.9×10^−16^). Nex20-treated NCC continued to move during the time course, though their speed was markedly reduced (31.84±13.1 µm/h; mean±s.d.) compared to vehicle (75.4±26.7 µm/h; Fig. 6D; *P*=4.0×10^−16^). In comparing the overall straightness (displacement of a cell from first to last frame/total length the cell travels), Nex20 NCC tracks showed little forward progress (Fig. 6B; Movie 6) and were significantly less straight (0.13±0.07; mean±s.d.) than vehicle (0.17±0.06; Fig. 6E; *P*=1.4×10^−3^). Finally, when CD63–pH electroporated neural folds were cultured overnight, compared to vehicle (Fig. 6F), Nex20-treated NCC showed a dramatic loss of CD63–pH-positive small vesicles (28±21 for Nex20 versus 129±75 for vehicle; mean±s.d.) immediately surrounding the cells (Fig. 6G,H, *P*=6.6×10^−9^), providing stark visual evidence that Nex20 blocked NCC exosome secretion.

## DISCUSSION

Our data show that NCCs release exosomes (Figs 1 and 4) and deposit migrasomes (Figs 2, 3), and that NCC exosome secretion is necessary to maintain directional movement (Fig. 6) of polarized cranial migratory NCCs (Fig. 5). Importantly, we demonstrate that NCCs produce exosomes and migrasomes normally, without Tspan overexpression. In conjunction with thin cellular bridges (McKinney et al., 2011; Teddy and Kulesa, 2004) and gap junctions (Huang et al., 1998; Jourdeuil and Taneyhill, 2020), exosomes and migrasomes present a novel means for cell–cell communication and transport of signaling molecules or morphogens between migratory NCCs.

Metastatic cells produce extracellular vesicles to alter the activities of other cells by promoting EMT (Conigliaro and Cicchini, 2018; Xiao et al., 2016), conditioning the metastatic niche (Peinado et al., 2016) and influencing directional migration (Qiao et al., 2019; Steenbeek et al., 2018; Sung et al., 2021, 2020; Zomer et al., 2015). However, it is unclear whether the use of exosomes to achieve these ends represents a cancer-specific process, hijacking of normal cellular physiology or reuse of a developmental program. Indeed, melanoma cells reactivate neural crest pathways during oncogenesis (Capparelli et al., 2022; Kaufman et al., 2016; Rambow et al., 2018) and EMT (Gupta et al., 2005). We wondered whether signaling through extracellular vesicles could be another normal NCC mechanism dysregulated by cancer cells. By taking advantage of chick cranial neural fold cultures, we show that NCCs release extracellular vesicles that are necessary for their migration. This reveals the possibility that metastatic cells co-opt NCC use of extracellular vesicles to achieve their disastrous effects.

Avoiding the complexity of imaging these tiny structures between cells deep within the embryonic chick head enabled the present work, but our NCC culture system is not without its challenges. A major limitation of this system is that we cannot purify NCC extracellular vesicles. Conventional exosome isolation methods are not adaptable to embryo- or explant-sized fluid volumes (McGough and Vincent, 2016). Meanwhile, migrasome content has been evaluated using 20,000 zebrafish embryos (Jiang et al., 2019) or 30–80 confluent 15 cm cell culture plates (Zhao et al., 2019). Technology is constantly improving, and there are new methods designed for small volumes; however, these are not yet optimized for exosome purity, yield or cost (Shirejini and Inci, 2022). Because NCC numbers are low relative to media volumes in our cultures, the results we obtained in testing these new methods produced low yields and would have required pooling several purifications, leaving us unsure of what we were actually isolating. Importantly, we show that BODIPY ceramide is a novel migrasome marker (Figs 2, 3), and HNK-1, which often colocalizes with CD63–pH in fixed cells (Fig. 1G), might be a neural crest-specific marker of extracellular vesicles. These additional labels will support critical future efforts to define the contents of NCC-derived extracellular vesicles once it is possible to overcome the limitations of scale. It is also worth noting that although the vast majority of cells in our cultures are HNK-1 positive (Fig. 1F″), a small amount of non-neural ectoderm is unavoidably included in neural fold dissections. Importantly, NCC migration is unaffected by scraping away this residual neural fold after NCC emigration (Bronner-Fraser and Garcia-Castro, 2008; Cousin and Alfandari, 2018), which we did not do in this work to avoid inducing the release of apoptotic bodies or other membranous debris from damaged or dying cells (Kakarla et al., 2020). In addition, since NCCs migrate out to ∼1 mm from the explant, any exosomes released from a non-motile cell type must act at a distance and would be supplied by conditioned medium, which did not rescue Nex20 treatment. Thus, NCC migration requires NCC-derived exosomes and not extracellular vesicles from a minor contaminating cell type.

It is interesting to speculate on the contents and function of NCC extracellular vesicles. We imaged NCCs at the furthest extent of migration from the explant, and NCC-derived extracellular vesicles were deposited on the substratum (Figs 1D,E and 2) and taken up by nearby cells (Fig. 2D). This suggests that NCCs at the migratory front release exosomes to provide migration regulatory factors, such as matrix metalloproteinases, guidance cues or chemotactic signals, that recruit or guide trailing cells in the stream (McLennan et al., 2015b; SenGupta et al., 2021; Shellard and Mayor, 2019; Sung et al., 2021, 2020). Our inability to rescue the migratory phenotype by adding back exosome-enriched medium to Nex20-treated cultures suggests a crucial role for extracellular vesicles deposited on the substrate (Fig. 1D), a high local concentration of exosomes released near cells or a gradient of soluble extracellular vesicles, none of which will be recreated by conditioned media.

Like cancer cells and neutrophils, NCC exosomes might also carry cargo that regulate cellular adhesions. Cancer cells release integrin-laden exosomes required for autocrine focal adhesion formation (Sung et al., 2015) and paracrine pathfinding behaviors in nearby cells (Sung et al., 2020). Similarly, activated neutrophils exhibit exocytosis-dependent (and Nex20-sensitive) surface upregulation of adhesion molecules, including integrins (Johnson et al., 2016). Given these observations, it is notable that exosome release was required for neural folds to adhere to fibronectin, suggesting that NCC extracellular vesicles might also carry integrins. Supporting this idea, binding of integrin receptors to fibronectin anchors focal adhesions, which stabilize cell protrusions and provide traction to migratory cells (Doyle et al., 2022). Disrupting this would explain the change in shape (Fig. 5H,I) and motility (Fig. 6) of Nex20-treated NCCs and is consistent with a role for exosomes in fibronectin-based NCC adhesion, similar to what is seen in cancer cells (Sung et al., 2015). Unfortunately, it was unclear whether NCCs, like cancer cells (Sung et al., 2020), navigate towards adhesion-rich exosome deposits because cranial neural fold cultures produce a dense population of directionally migrating NCC that obscure the pathfinding behavior of any individual cell (Movie 2).

Finally, it is possible that NCC extracellular vesicle production facilitates rapid clearance of proteins that antagonize EMT. In chick cranial NCCs, such proteins could include Tspan18 (Fairchild et al., 2014; Fairchild and Gammill, 2013) and cadherin 6B (Coles et al., 2007). In support of this idea, during cranial NCC EMT, epithelial cadherin 6B is internalized into the endocytic pathway (Padmanabhan and Taneyhill, 2015), which generates exosomes (Sung et al., 2021). In addition, Tspan18 promotes migrasome formation (Huang et al., 2019), and Tspan-enriched microdomains contribute to the ceramide-dependent biogenesis of exosomes (Zhang et al., 2019). Thus, in addition to serving a signaling function, the release of extracellular vesicles by NCCs might be the final step to ‘take out the trash’ when cell surface contacts change dramatically during EMT.

Cancer exosome research, largely performed on cell lines, yields enormous promise for innovative drug delivery, identification of diagnostic biomarkers and discovery of cancer mechanisms (Soung et al., 2017; Tickner et al., 2014). It is crucial to support these efforts by revealing exosome function in normal physiological contexts where exosome release is not dysregulated (Kalluri and LeBleu, 2020). By imaging NCC emigrating from a neural fold explant one step removed from the embryo, we document embryonic NCC exosome and migrasome release and define exosomes as critical for NCC migration, a normal developmental process related to metastasis (Theveneau and Mayor, 2012).

## MATERIALS AND METHODS

### Embryos

Fertilized chicken eggs (*Gallus gallus*, strain ISA Brown, sex not determined) were acquired from a local hatchery and incubated in a humidified incubator (G. Q. F. Manufacturing; Savannah, GA, USA) at 38°C. Embryos were staged by counting somite pairs or by comparison to Hamburger and Hamilton (1992) and incubated to embryonic ages between gastrulation and Hamburger and Hamilton stage 9 (7 somites). All animal experiments were performed according to approved guidelines.

### DNA construct and electroporation

pCMV-CD63-pHluorin was obtained from the Verweij laboratory (Department of Biology, Utrecht University, The Netherlands; Verweij et al., 2018). Late gastrula Hamburger and Hamilton stage 4–5 embryos were electroporated as previously described (Gammill et al., 2019). Briefly, embryos were isolated from the yolk by adhering to Whatman filter paper, rinsed in chick Ringer's saline, and put into an electroporation cuvette with a 4 mm gap. Embryos were bilaterally injected with 1 µg/µl pCMV-CD63-pHluorin from the ventral side into the subvitelline space adjacent to neural crest precursors, then electroporated using five square-wave 7 V 50 ms pulses with 100 ms gaps. Electroporated embryos were cultured on agar–albumin plates and incubated to the stage indicated.

### Neural crest cultures

Glass coverslips (Carolina Biological Supply) in tissue culture wells (for fixed imaging) or 35 mm dishes with 20 mm microwell glass inserts (Cellvis; for live imaging) were coated with 10–100 μg/ml fibronectin (Thermo Fisher Scientific) diluted in Ringer's saline for at least 1 h. Cranial neural folds were dissected from 3–7 somite stage chick embryos as previously described (Jacques-Fricke et al., 2022) and plated in neural crest culture media made up of Leibovitz's L-15 medium, 1% L-glutamine and 0.1% penicillin/streptomycin (Thermo Fisher Scientific) supplemented with 10% fetal bovine serum (FBS; Thermo Fisher Scientific) and 10% chick embryo extract (Bronner-Fraser and Garcia-Castro, 2008). After incubation for 4–24 h at 37°C, cultures were fixed for 15 min with 4% paraformaldehyde.

### Immunohistochemistry

Fixed cultures were blocked in 10% FBS plus PBS and 0.1% Triton X-100, and stained with primary antibodies for HNK-1 (DSHB, Iowa City, IA; 1:250 mouse monoclonal, clone 3H5) or Hsc70 (Abcam, 1:75 rat monoclonal, clone 1B5), followed by Alexa Fluor 568-conjugated goat anti-rat-IgG (Life Technologies, 1:250) or RRX-conjugated goat anti-mouse IgM (Jackson ImmunoResearch Laboratories, Inc, 1:250) secondary antibodies. HNK-1 was raised against a chick epitope and has confirmed positive reactivity in chick by the Developmental Studies Hybridoma Bank with many publications (https://dshb.biology.uiowa.edu/3H5). Hsc70 was confirmed by Abcam to have positive reactivity in chick (https://www.abcam.com/hsc70-antibody-1b5-ab19136.html). Cells were counterstained with Oregon Green or Alexa Fluor 647 phalloidin (both Thermo Fisher Scientific). Coverslips were mounted on glass slides using PermaFluor mounting medium with 1 μg/ml DAPI (both Thermo Fisher Scientific).

### Live-cell dyes

Neural crest cultures were incubated with BODIPY TR Ceramide (Thermo Fisher Scientific) according to the manufacturer's instructions. Briefly, cultures were incubated with 5 µM BODIPY ceramide-BSA (Thermo Fisher Scientific) diluted in cold Hanks’ balanced salt solution (HBSS) for 15 min at 4°C before washing with cold HBSS and re-incubating at 37°C for 2 h prior to imaging. Hoechst 33342 (Thermo Fisher Scientific) was added to HBSS at a concentration of 1 μg/ml and cultures were re-incubated at 37°C for 10 min, washed with HBSS, and then fresh medium was added prior to imaging. SYTO14 Green (Thermo Fisher Scientific) or WGA CF488A or CF633 (Biotium) was added to neural crest medium at a concentration of 2.5–5 µM for 10 min at 37°C. Cells were then washed with PBS, and fresh medium was added prior to imaging.

### Exosome depletion of neural crest culture medium and C8161 cell culture

Neural crest culture medium was prepared and subjected to ultracentrifugation as described in Théry et al. (2006). Briefly, culture media was filtered with a 0.22 µm filter (Millipore Sigma), transferred to 10.4 ml polycarbonate round bottom tubes (Beckman Coulter) and spun at 4°C for 24 h at 100,000 ***g*** in a 70.1 Ti rotor (Beckman Coulter) to pellet exosomes. Depleted medium (supernatant) was removed and frozen in single-use (3 ml) aliquots. This medium was used in neural fold and C8161 cell cultures for NTA analysis. C1861 cells are derived from a human abdominal wall metastasis (Welch et al., 1991) and were obtained from the Stowers Institute for use as a positive control; they were not authenticated or tested for contamination. C8161 cells were plated in various numbers (25,000, 50,000 or 75,000) in wells of a 24-well plate in exosome-depleted NCC medium and incubated at 37°C for 24 h prior to NTA analysis.

### Nanoparticle tracking analysis

Image capture and analysis were performed using a NanoSight LM-10 equipped with a 400 nm laser (Malvern Panalytical). Cell culture supernatants were diluted 1:250 or 1:500 with 0.22 µm filtered PBS prior to imaging for optimal visualization of single vesicles. 3K/4K Series Particle Counter Standard beads (Thermo Fisher Scientific) were run prior to samples in order to calibrate the system for 0.1 µm particles and act as a positive control. Image capture was set for five repeated captures at 45 s intervals. Data was analyzed using NanoSight software (Malvern Panalytical) and exported to Excel .csv files (Microsoft). Experiments were performed in three biological replicates.

### Inhibitor assays

Nexinhib20 (Tocris) was resuspended as a stock at 50 mM in ethanol, further diluted at 2.5 mM in ethanol or methanol (with equivalent results) and used at 2.5 µM (Johnson et al., 2016) in exosome-depleted neural crest culture medium. Diluted inhibitor or vehicle control was added to neural crest cultures after neural folds had been incubated for 3–4 h at 37°C to allow them to adhere to the fibronectin substrate.

### Cell death assay

An *In Situ* Cell Death Detection Kit, TMR Red (Roche) was utilized on fixed cells according to the manufacturer's instructions with Label Solution diluted 1:5 with PBS to reduce TMR Red signal background. DAPI and TMR channels were captured using a 20× objective in six to eight images around the edges of each culture where the NCC had migrated the furthest (two cultures per experiment, three biological replicates). Images were uploaded to ImageJ (NIH), brightness and contrast adjusted, and nuclei were counted manually (DAPI, all nuclei; TMR-positive, fragmented nuclei) to calculate the percentage of dying cells. Outcomes were averaged and significance determined by an unpaired one-tailed Student's *t*-test with Bonferroni correction for multiple comparisons.

### Microscopes and image acquisition

#### Fixed cell imaging – confocal microscopy

Images were acquired on an AxioObserver equipped with an LSM710 confocal scan head controlled by ZEN 3.0 SR software (Zeiss). Plan-Apochromat 10×/0.45NA, Plan-Apochromat 20×/0.8 M27, Plan-Apochromat 40×/0.95 Korr M27, and alpha Plan-Apochromat 100×/1.46 Oil DIC M27 objectives (all Zeiss) were used, and the following excitation and emission settings were used in sequential (track) mode: DAPI 405 nm excitation, 410–495 nm emission; GFP or Alexa Fluor 488, 488 nm excitation, 493–578 nm emission; TMR or Rhodamine Red-X, 561 nm excitation, 568–646 nm emission; Alexa Fluor 647, 633 nm excitation; 638–755 nm emission. Pixel size was set at 1.38 µm and the confocal aperture at 90 µm. Images were scanned at a pixel dwell time of 0.79 µs with four times line averaging.

#### CD63–pH live-cell imaging – spinning disk confocal microscopy

Images were acquired with an Axio.ObserverZ1 motorized microscope (Zeiss) equipped with a CSU-X1 confocal scan head (Yokogawa) controlled by ZEN software (2.6; Zeiss). A C-ApoCHROMAT 63×/1.20 NA water immersion objective (Zeiss) was used with the following excitation and emission settings: CD63–phluorin, 488 nm excitation, 503–538 nm emission (50 ms exposure time). Images were collected with a QuantEM:512SC camera (Photometrics) and an image pixel size of 0.212×0.212 µm (*xy*). Images were collected every 5 min for 3 h and the focal plane was maintained with Definite Focus (Zeiss). Samples were maintained at 37°C with saturating humidity.

#### Inhibitor live-cell imaging – spinning disk confocal microscopy

Cells were imaged at 1 to 4 Hz on a 3i Marianas spinning disk confocal (Intelligent Imaging Innovation) with a Plan-Apochromat 20×/0.8 NA Ph2, 0.323 µm pixel size (Zeiss) or 63× Plan-Apochromat 63×/1.4 NA oil DIC, 0.212 µm pixel size (Zeiss) objective. The sample temperature was maintained at 37–38°C in a heated sample chamber. Samples were illuminated in DIC using a CSU-X1 M1 spinning disk (Yokogawa) and captured with an EMCCD camera (Evolve). 5–10 *Z*-sections of 0.4–0.5 µm were taken with a 50–250 ms exposure with 10–40% laser power. Cells were imaged every 2 min or longer for 4–6 h depending on experiment.

### Quantification of fixed neural crest cultures

Images were imported into NIH ImageJ version 1.53j. After thresholding, the ‘Analyze Particles’ function was used to calculate the area occupied by migrating cells and total number of cells migrated in neural fold cultures at low magnification, or aspect ratio (the ratio between the length of a cell and its height) and circularity [circularity=4π(area/perimeter^2^)] of NCC at high magnification. The significance of the effects were evaluated by an unpaired one-tailed Student's *t*-test statistical analysis in Excel (Microsoft). Images were adjusted in Photoshop (Adobe).

### Quantification of live neural crest cultures

Maximum intensity projections of recorded images were exported into ImageJ for analysis. Images were uploaded into MTrackJ plugin (Image Science, Erik Meijering), where five individual cells from each video (three videos per condition per experiment) were manually tracked. Data was measured in MTrackJ to give output on length, displacement from first to final frame (straightness) and speed (µm/h). Three biological replicates were performed. For vesicle counting, *z*-stack images were taken of 25 fields of view from two folds per experiment. Three biological replicates were performed. Maximum intensity projections were generated of images and processed using the Analyze Particle function in ImageJ. For quantifying migrasome diameter, 111 migrasomes positive for BODIPY ceramide and WGA or SYTO14 were manually counted in ImageJ. Significance was determined by unpaired one-tailed Student's *t*-test statistical analysis in Excel (Microsoft). Images were adjusted in Photoshop (Adobe).

## Supplementary Material

Supplementary information
