# Supplementary material for: Chick cranial neural crest cells release extracellular vesicles that are critical for their migration
Source: J Cell Sci. 2022 Jun 28;135(12):jcs260272. doi: 10.1242/jcs.260272 (PMC9270958; doi:10.1242/jcs.260272)
Supplement: Supplementary information [file joces-135-260272-s1.pdf]

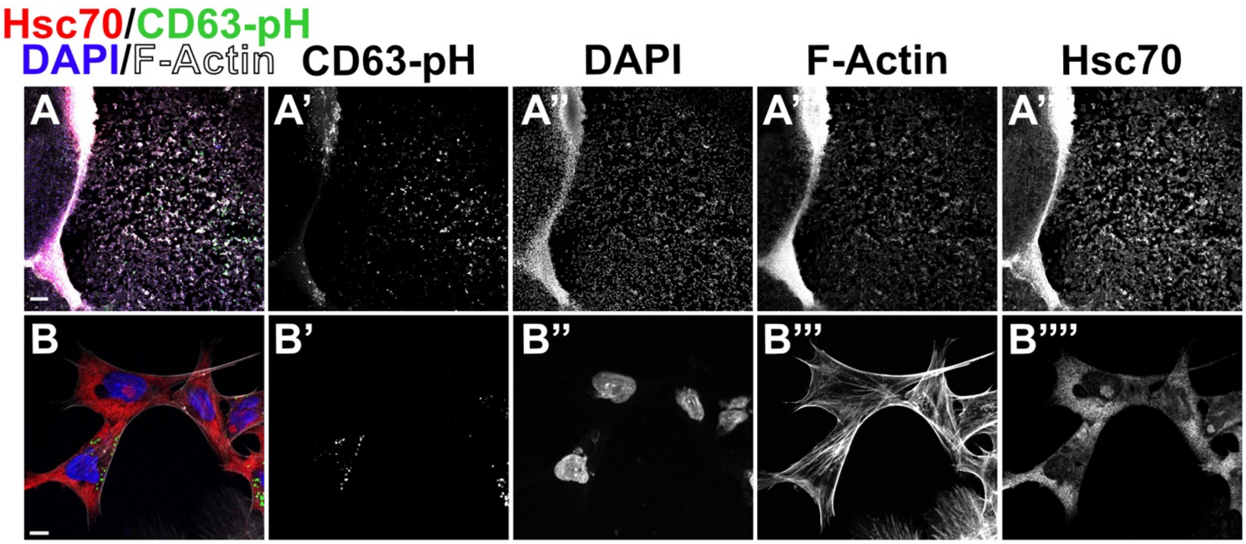

Fig. S1. Neural crest cells express the exosome marker Hsc70. (A, B) Fixed neural fold cultures (A-A””) and individual neural crest cells (B-B””) expressing CD63-pH (exosomes, green) stained with DAPI (nucleus, blue), F-actin (cytoskeleton, white), and Hsc70 (cytoplasmic exosome marker, red). Scale bars: A, 100 µm; B, 10 µm.

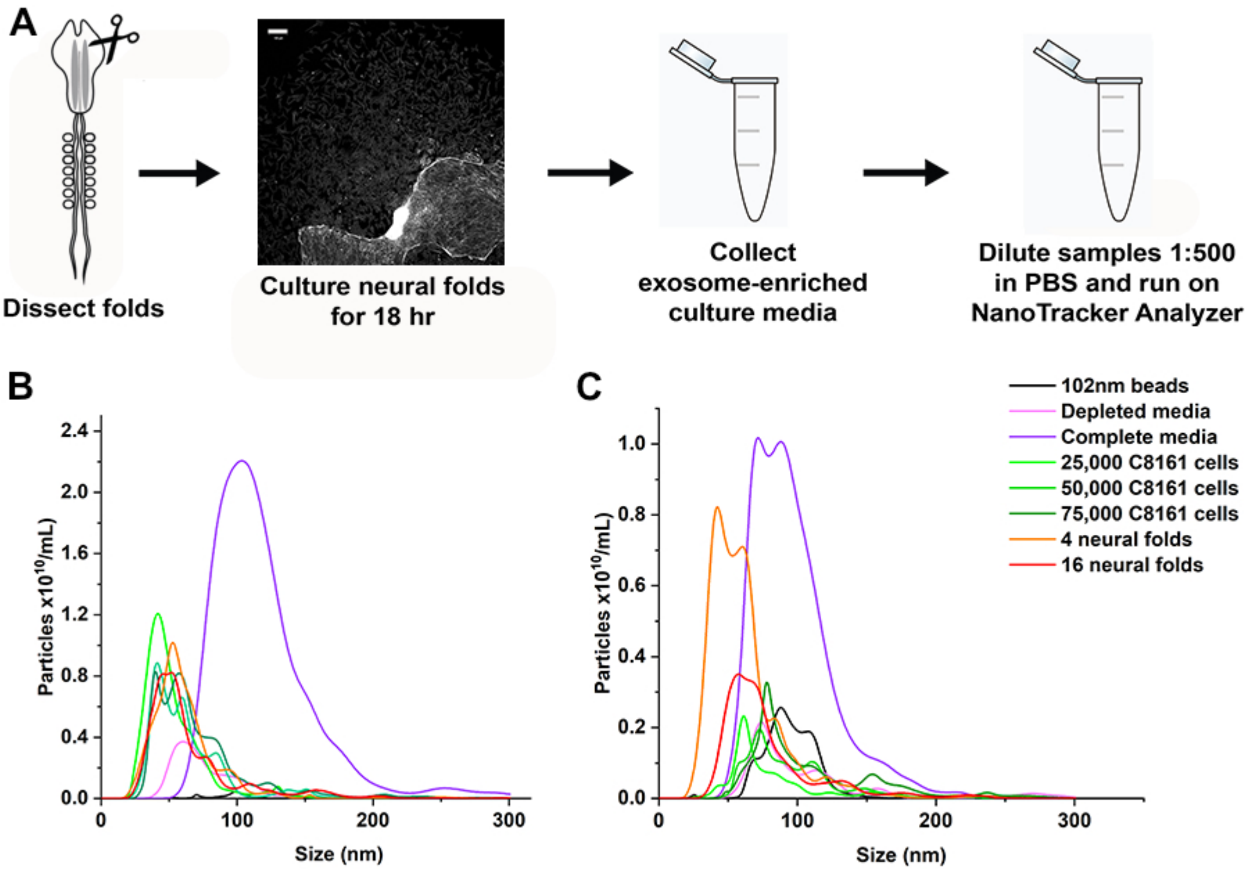

Fig. S2. Experimental plan of NTA sample preparation and additional experimental replicates from neural crest cell NTA analysis. (A) Schematic of sample preparation for Nanoparticle Tracking Analysis (NTA) of neural folds cultured in exosome-depleted media. Scale bar: 10 µm **(B, C)** NTA of particle size (nm) versus concentration (particles x 10^{10}/mL) shown as merged graphs of all experimental conditions including media before or after culture with neural folds or C8161 cells in two additional biological experiments. Traces are averaged from 5 replicate video captures for each. These graphs illustrate the experiment-to-experiment variability of data acquisition by NTA while still supporting the stated conclusions.

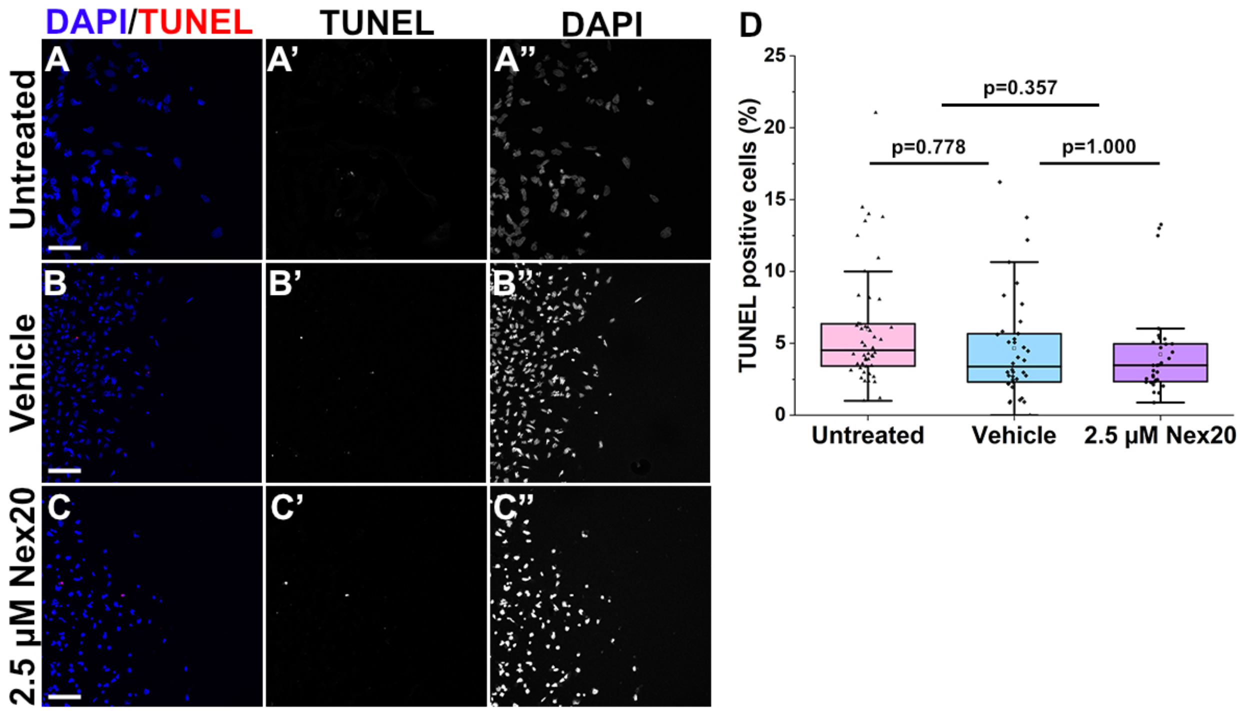

Fig. S3. Nex20 treatment does not cause cell death in cranial neural crest cells. **(A-C)** Neural crest cells stained for TUNEL-TMR (nuclear DNA damage, red) and DAPI (nucleus, blue) in untreated (A-A"), vehicle (B-B") and 2.5 μM Nex20 (C-C") treated cultures. Scale bars: A, 50 μm; B, C, 100 μm. **(D)** Box and whisker plot of untreated, vehicle and 2.5 μM Nexinhib20-treated culture conditions. Bars represent the percentage of DAPI-positive nuclei from neural crest cells imaged and manually counted. Neural crest cells were identified as TUNEL positive from co-localization of TUNEL-TMR signal in DAPI-positive nuclei and shown as a percentage of total cells counted (untreated n=2041; vehicle n=5010; 2.5 μM Nex20 n=4231). Student's t-test was performed between untreated and vehicle (p=0.778), vehicle and 2.5μM Nex20 (p=1.000), and untreated and 2.5 μM Nex20 (p=0.357) conditions.

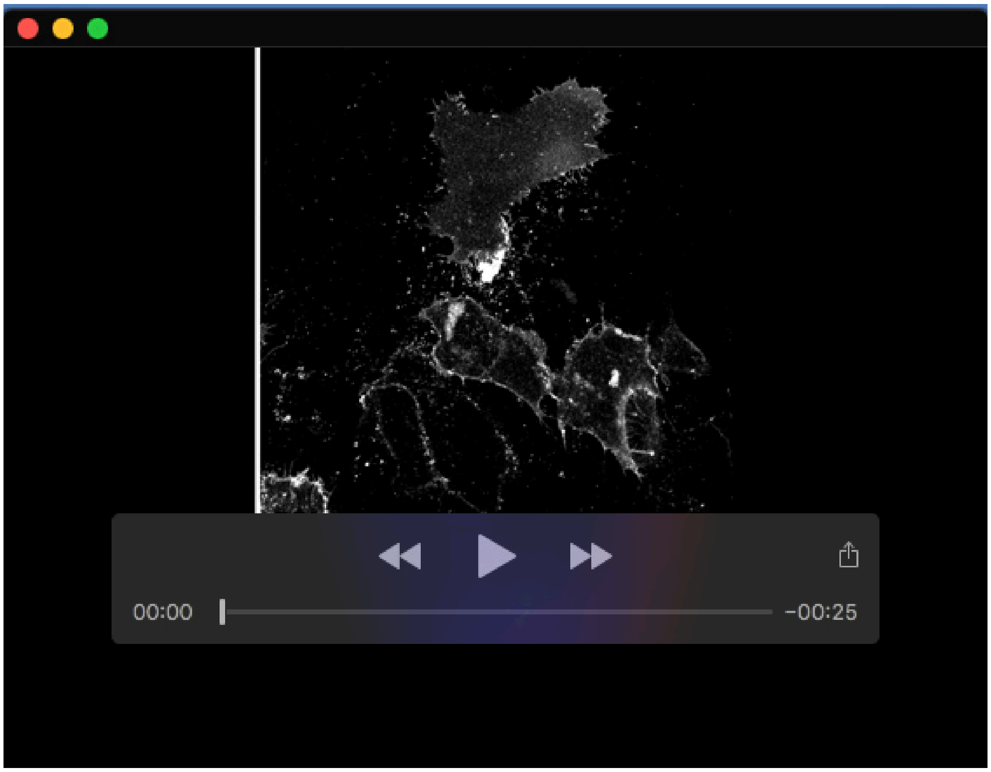

Movie 1. CD63-pHluorin-positive cranial neural crest cells release bright extracellular puncta (related to Figure 1C'). Time lapse of CD63-pHluorin extracellular puncta released from neural crest cells. Single-plane confocal images captured by an LSM710 confocal microscope with an interval of 60 seconds for 15 minutes.

Journal of Cell Science • Supplementary information

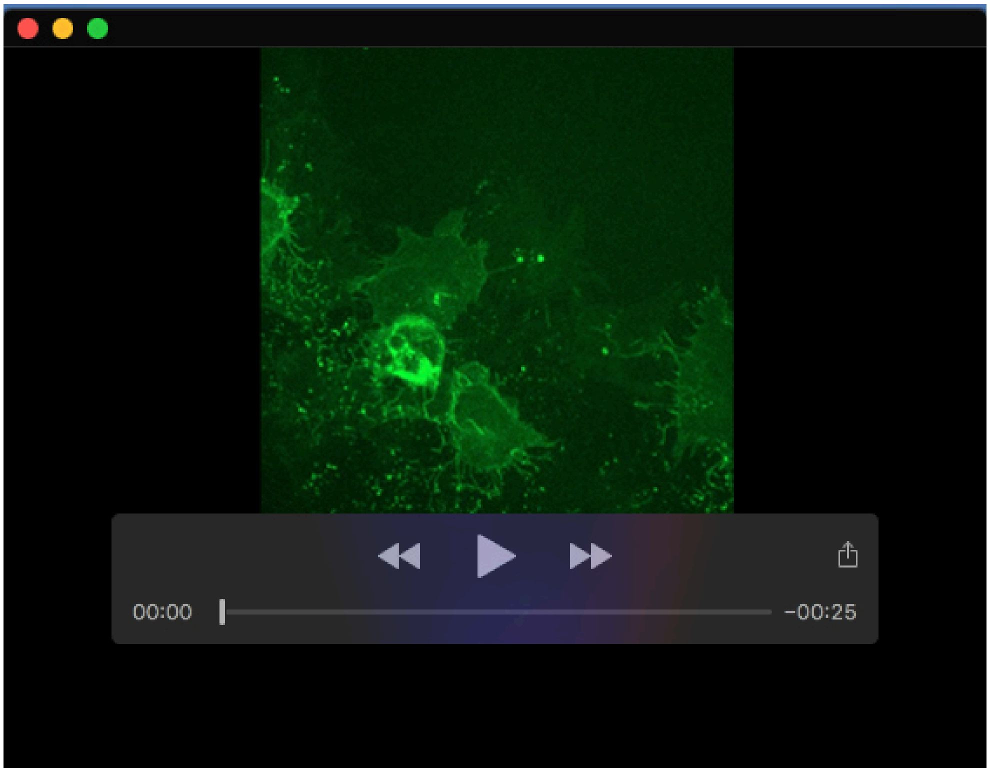

Movie 2. Migrating CD63-pHluorin-positive cranial neural crest cells deposit exosome trails (related to Figure 1G). Time lapse of migratory CD63-pHluorin neural crest cells. Maximum intensity projection confocal images captured by a spinning disk Axio.ObserverZ1 confocal microscope with an interval of 2 minutes for 3 hours.

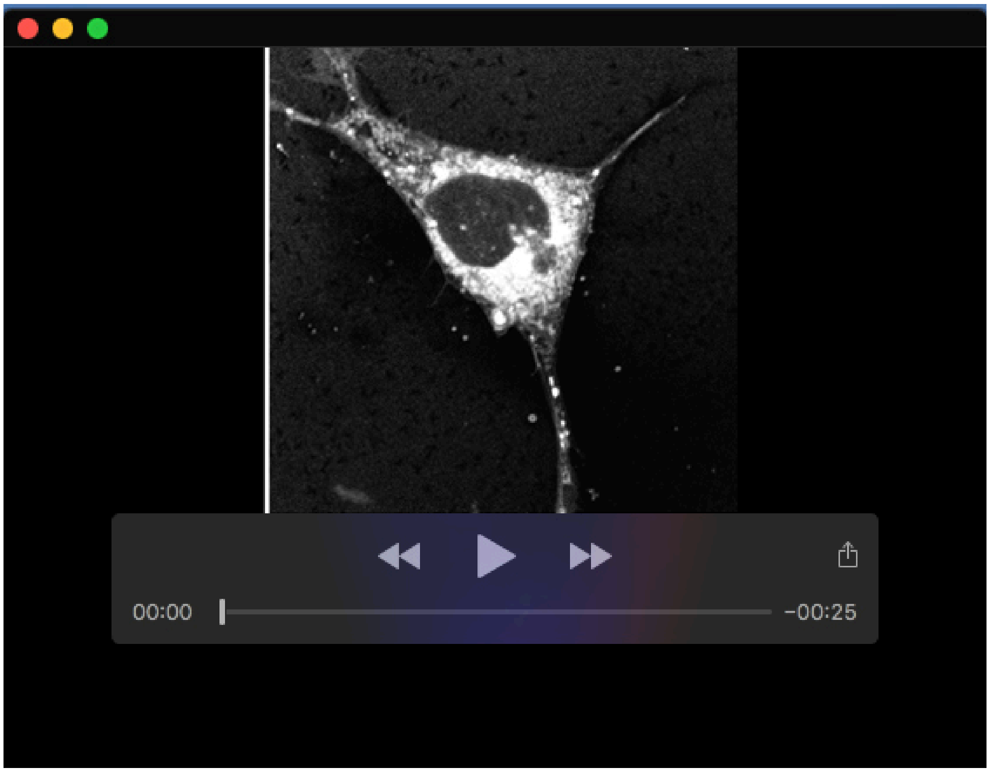

Movie 3. BODIPY ceramide-labeled neural crest cell contains fluorescent multivesicular bodies (related to Figure 2A). Time lapse of single BODIPY ceramide-labeled neural crest cell containing moving fluorescent multivesicular bodies. Maximum intensity projection confocal images captured by a LSM710 confocal microscope with an interval of 30 seconds for 10 minutes.

Journal of Cell Science • Supplementary information

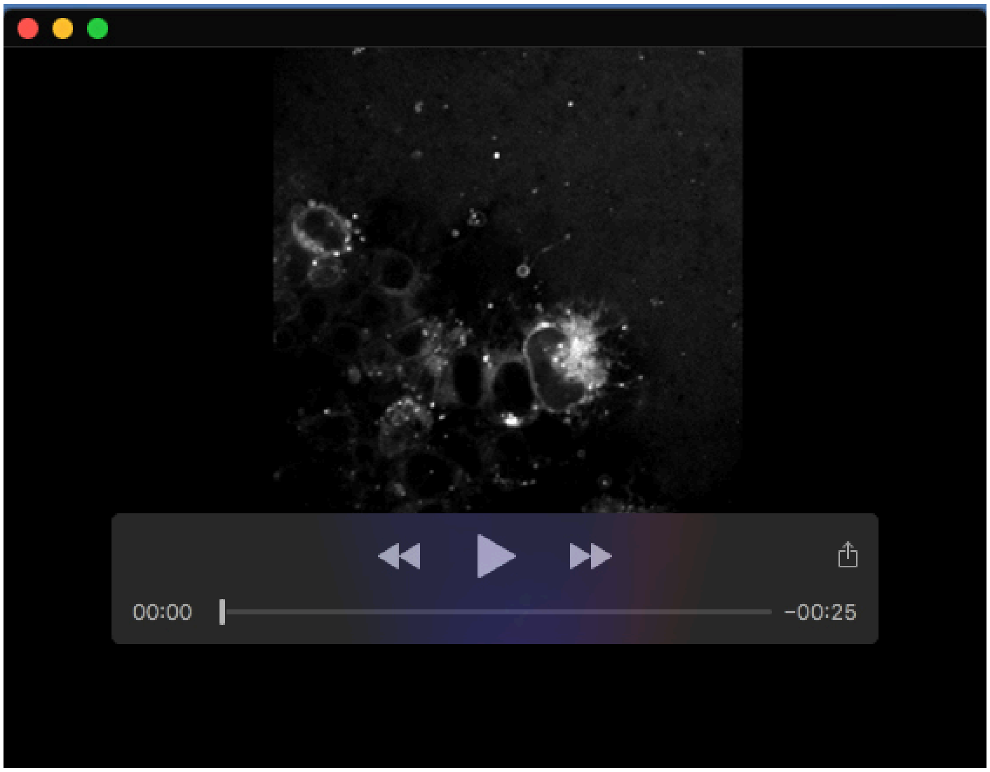

Movie 4. BODIPY ceramide-labeled migrasome taken up by migrating neural crest cell (related to Figure 2D). Time lapse of a BODIPY ceramide-labeled migrasome being taken up by a BODIPY ceramide-labeled neural crest cell. Maximum intensity projection confocal images captured by spinning disk 3i Marianas confocal microscope with an interval of 2 minutes for 4 hours.

Journal of Cell Science • Supplementary information

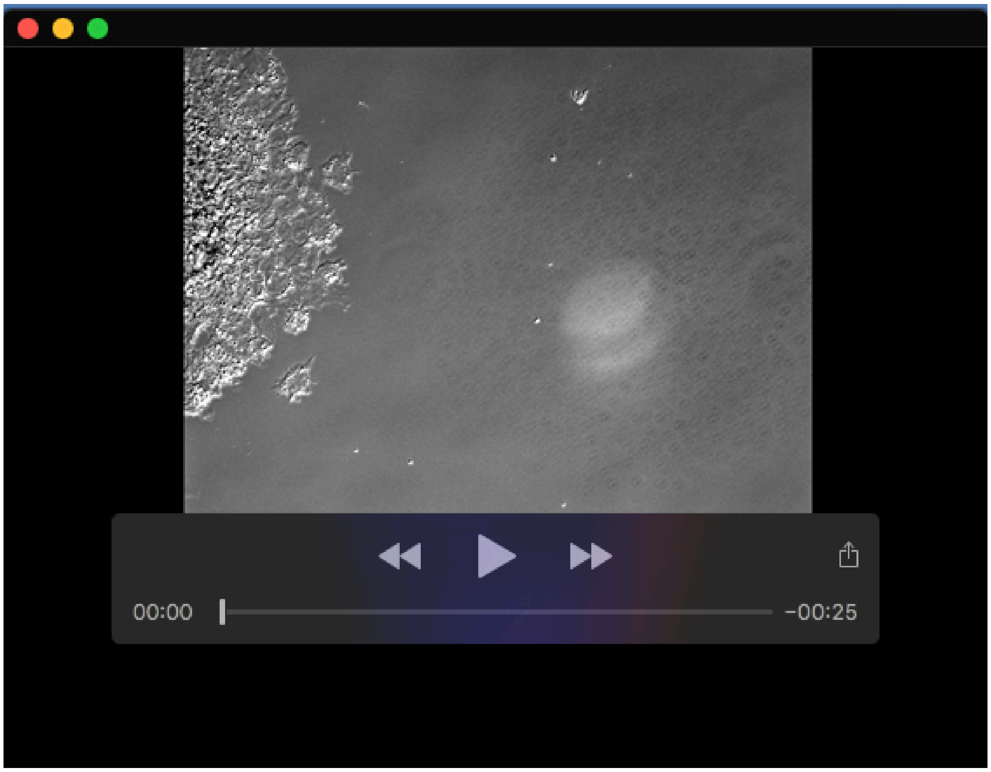

Movie 5. Vehicle-treated neural crest cells migrate away from neural fold (related to Figure 5A). Time lapse of a field of neural crest cells treated with vehicle control migrating away from the neural fold. Maximum intensity projection confocal DIC images captured by spinning disk 3i Marianas confocal microscope with an interval of 2 minutes for 6 hours. Video compression to 20 frames per second.

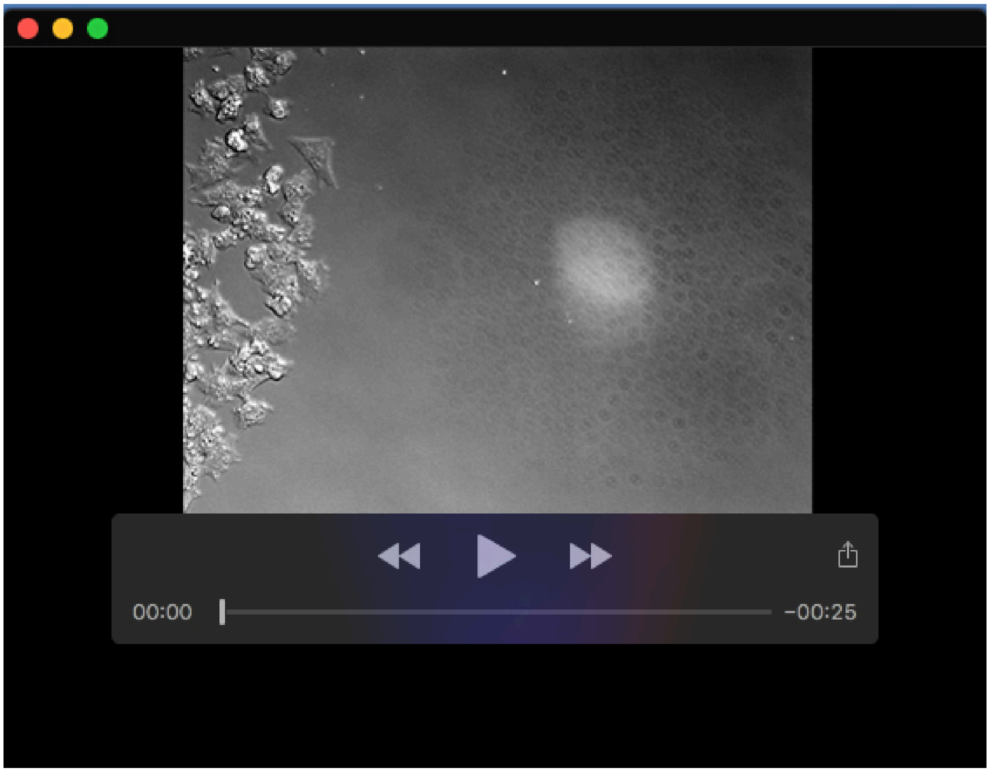

Movie 6. Nex20-treated neural crest cells fail to migrate away from neural fold (related to Figure 5A). Time lapse of a field of neural crest cells treated with Nex20 failing to migrate away from the neural fold. Maximum intensity projection confocal DIC images captured by spinning disk 3i Marianas confocal microscope with an interval of 2 minutes for 6 hours. Video compression to 20 frames per second.
